# Time-Dependent Probability Density Functions and Attractor Structure in Self-Organised Shear Flows

**DOI:** 10.3390/e20080613

**Published:** 2018-08-17

**Authors:** Quentin Jacquet, Eun-jin Kim, Rainer Hollerbach

**Affiliations:** 1 Department of Applied Mathematics, University of Leeds, Leeds LS2 9JT, UK; 2 ENSTA ParisTech Université Paris-Saclay, 828 Boulevard des Maréchaux, 91120 Palaiseau, France; 3 School of Mathematics and Statistics, University of Sheffield, Sheffield S3 7RH, UK

**Keywords:** self-organisation, shear flows, coherent structures, turbulence, stochastic processes, Langevin equation, Fokker-Planck equation, information length

## Abstract

We report the time-evolution of Probability Density Functions (PDFs) in a toy model of self-organised shear flows, where the formation of shear flows is induced by a finite memory time of a stochastic forcing, manifested by the emergence of a bimodal PDF with the two peaks representing non-zero mean values of a shear flow. Using theoretical analyses of limiting cases, as well as numerical solutions of the full Fokker–Planck equation, we present a thorough parameter study of PDFs for different values of the correlation time and amplitude of stochastic forcing. From time-dependent PDFs, we calculate the information length (L), which is the total number of statistically different states that a system passes through in time and utilise it to understand the information geometry associated with the formation of bimodal or unimodal PDFs. We identify the difference between the relaxation and build-up of the shear gradient in view of information change and discuss the total information length ($L_{\infty }=L\left(t\to \infty \right)$) which maps out the underlying attractor structures, highlighting a unique property of $L_{\infty }$ which depends on the trajectory/history of a PDF’s evolution.

## 1. Introduction

Many systems in nature and laboratories are far from equilibrium, constantly changing in time and space and exhibiting very complex behaviour. Examples include turbulence in astrophysical and laboratory plasmas, the stock market, and biological ecosystems. Despite having apparently different manifestations of complexity, these systems have much in common and are often governed by similar nonlinear dynamics. In particular, an ‘ordered’ collective behaviour (e.g., in the form of coherent structures) emerges on the macroscale out of complexity as a novel consequence of self-organisation. For example, in the laboratory, in geophysical and astrophysical systems, coherent structures such as large-scale shear flows (such as zonal flows and streamers in laboratory plasmas, in the atmosphere and oceans, and in giant planets) and differential rotations in the Sun and other stars emerge from small-scale turbulence. There is overwhelming evidence from laboratory experiments, observations, and computational studies that these coherent structures play an absolutely critical role in determining the level of transport in the flow.

In particular, one crucial effect of shear flows is the suppression of transport in the direction orthogonal to the flow (the shear direction) by shear-induced enhanced dissipation [1,2,3,4,5,6,7,8,9,10,11]. This occurs as a shear flow distorts fluid eddies, accelerates the formation of small scales, and dissipates them when molecular diffusion becomes effective on small scales. This turbulence regulation leads to the formation of a transport barrier where transport is significantly reduced locally, providing one of the crucial mechanisms for controlling the mixing and transport in a variety of systems. Important examples include (i) the low-to-high (L-H) transition (or internal transport barrier formation), during which a system undergoes a remarkable, spontaneous transition to a more ordered state, despite the increase in free energy (e.g., [3,4,5]); (ii) equatorial winds and polar vortices [12] (azimuthal flows in the east–west direction) which have long been known to reduce transport, acting as a transport barrier in the latitudinal direction [13]; (iii) transport barrier due to shear layers [14] in oceans which is called shear sheltering; and (iv) the solar tachocline—the boundary layer between the stable radiative interior and unstable convective layer which has a strong radial differential rotation—which can also act as a transport barrier, leading to weak anisotropic turbulence and mixing [5,7]. Our theoretical predictions of turbulent quenching in different systems have been confirmed by various numerical simulations (e.g., refs. [15,16]).

The foregoing statements underscore the importance of self-regulation between small-scale fluctuations and large-scale shear flows. We proposed a one-dimensional (1D) continuous model of self-organised shear flow [17] by extending a prototypical sand-pile model which evolves in discrete time. Specifically, we considered the formation of a shear flow driven by a short-correlated (white-noise) random forcing, where the shear gradient increases until it becomes unstable according to the stability criterion. For instance, in a strongly stratified medium, the stability is determined by the Richardson criterion: fluctuations on small scales (or internal gravity waves) amplify a shear gradient and thus, act as a forcing until the gradient exceeds the critical value given by the Richardson criterion, $R={\left(A/N\right)}^{2}>R_{c}={\left(A_{c}/N\right)}^{2}=1/4$. Here, *N* is the buoyancy frequency due to the restoring force (buoyancy) in a stably stratified medium, and *A* is the shear gradient with the critical value $A_{c}$. When unstable, the shear flow then relaxes its gradient and generates small-scale fluctuations, and this relaxation was modelled by nonlinear (cubic) diffusion; the shear gradient then grows again when small-scale turbulence becomes sufficiently strong to drive a shear flow. The same cycle repeats itself, exhibiting continuous growth and damping. This highlights that a self-organised state is never stationary in time, but involves persistent fluctuations.

The extension of refs. [17,18] solved a stochastic differential equation with a fourth-order stochastic Runga–Kutta method for Gaussian coloured noise in 1D and showed the transition from an unimodal stationary Probability Density Function (PDF) to a bimodal stationary PDF when the correlation time of a random forcing exceeds a critical value. The mean shear gradient is zero for a unimodal PDF, while its non-zero value represents the critical shear gradient around which a shear gradient continuously grows and damps through the interaction with fluctuations. The transition from a unimodal to bimodal PDF represents the formation of a non-zero mean shear gradient, or the formation of jets. Interestingly, In ref. [18], we found similar results in a 0D model and 2D hydrodynamic turbulence. In particular, the 2D results showed that a shear flow evolves through the competition between its growth and damping due to a localized instability, maintaining a stationary PDF, and that the bimodal PDF results from a self-organising shear flow with a linear profile.

The purpose of this paper is to investigate the evolution of a time-dependent PDF to understand how a given initial (global) shear gradient modelled by a narrow PDF relaxes into a bimodal or unimodal stationary PDF. We are particularly interested in understanding the information geometry associated with this process. Our information geometry theory is based on the Fisher metric [19] extended to time-dependent problems. (Note that we use information about statistically different states, refraining from the debate on the exact definition of information [19,20]). We recall that for a Gaussian PDF whose evolution is described by the movement of a peak and the change in its width, the uncertainty measuring the mean value of *x* is set by the standard deviation. Two PDFs with the same standard deviation would differ by one statistical state when their mean values differ by the standard deviation (e.g., see ref. [21]). To formalise this idea to quantify the information change associated with the time evolution of PDFs [22,23,24,25,26,27,28,29,30,31,32], we define an infinitesimal distance at any time by comparing two PDFs at adjacent times and sum these distances. The total distance gives us the number of statistically different states that a system passes through in time and is called the information length (L). While the detailed derivation of L and its applications are given in refs. [22,23,24,25,26,27,28,29,30,31,32], it is useful to highlight that L is a measure of the total elapsed time in units of a dynamical timescale for information change. To show this, we define the dynamical time (τ(t)) [22,23,24,25,26,27,28,29,30] as follows:

(1) $$E\equiv \frac{1}{{\left[\tau \left(t\right)\right]}^{2}}=\int \frac{1}{p(x,t)}{\left[\frac{\partial p(x,t)}{\partial t}\right]}^{2}\;dx.$$

Here, τ(t) is the characteristic timescale over which the information changes. Having units of time, τ(t) quantifies the correlation time of a PDF. Alternatively, 1/τ quantifies the (average) rate of change of information in time. L(t) is then defined by measuring the total elapsed time (*t*) in units of τ as

(2) $$L\left(t\right)=\int_{0}^{t}\frac{dt_{1}}{\tau (t_{1})}=\int_{0}^{t}\sqrt{\int dx\frac{1}{p(x,t_{1})}{\left[\frac{\partial p(x,t_{1})}{\partial t_{1}}\right]}^{2}}\;dt_{1}.$$

L(t) measures the cumulative change in p(x,t), and depends on the intermediate states that a system evolves through between times 0 and *t*. Thus, it is a Lagrangian quantity (unlike entropy or relative entropy) which depends on the time history of p(x,t), uniquely defined as a function of time *t* for a given initial PDF. L represents the total number of statistically distinguishable states that a system evolves through, providing a very convenient methodology for measuring the distance between p(x,t) and p(x,0) continuously in time for a given p(x,0). References [22,23,24,25,26,27,28,29,30,31,32] showed that $L_{\infty }$ is a new diagnostic for understanding a dynamical system and for mapping out an attractor structure. In particular, $L_{\infty }$ captures the effect of different deterministic forces through the scaling of $L_{\infty }$ against the peak position of a narrow initial PDF. For a stable equilibrium, the minimum value of $L_{\infty }$ occurs at the equilibrium point. In comparison, in the case of a chaotic attractor, $L_{\infty }$ exhibits a sensitive dependence on initial conditions like a Lyapunov exponent.

In this paper, we investigate the evolution of a shear gradient (*x*) starting from a relatively narrow PDF (p(x,0)) with an initial mean value of $x_{0}$ which represents the mean value of an initial shear gradient. For a unimodal stationary PDF, the mean shear gradient decreases to zero in the long time limit, while for a bimodal stationary PDF with a peak of $\pm x_{*}$, the case of $x_{0}>x_{*}$ models the relaxation of an initial super-critical gradient ($x_{0}$) to the critical value ($x_{*}$), and the case of $x_{0}<x_{*}$ models the build-up of the gradient from a subcritical initial value to the critical value ($x_{*}$). We are interested in the information changes in these processes and in identifying the differences between the relaxation and build-up of the shear gradient in view of these information changes and in mapping out an attractor structure by using L.

The remainder of this paper is organised as follows. We introduce our model and provide analytical solutions of time-dependent PDFs in limiting cases in Section 2. In order to systematically undertake a numerical study, in Section 3, we first provide a detailed discussion on stationary PDFs for different parameter values to determine the parameter space for unimodal versus bimodal PDFs. Section 4 provides numerical solutions for time-dependent PDFs and L. The discussion and conclusions are found in Section 5.

## 2. Model

In this section, we introduce our model and provide analytical solutions for time-dependent PDFs in limiting cases. As noted in Section 1, given the universality of self-organisation in 0D, 1D, and 2D models and the challenge of the computation of time-dependent PDFs, we utilised a 0D model to facilitate the calculation of PDFs. Our 0D model is based on the cubic process for a stochastic variable (*x*) (e.g., representing a shear gradient). Specifically, we considered *x* driven by a finite correlated forcing (*f*), governed by the following Langevin equations

(3) $$\begin{array}{ccc} \partial_{t}x & = & -\left(ax+bx^{3}\right)+f\equiv -g\left(x\right)+f, \end{array}$$

(4) $$\begin{array}{ccc} \partial_{t}f & = & -\gamma f+\xi . \end{array}$$

Here, $g\left(x\right)=ax+bx^{3}$; a,b≥0 are constants; ξ is a stochastic noise with a short correlation time with the correlation function

(5) $$\langle \xi \left(t\right)\xi \left(t^{\prime }\right)\rangle =2D\delta \left(t-t^{\prime }\right).$$

The highest cubic nonlinearity in our 0D model mimics a nonlinear cubic diffusion in the 1D model in refs. [17,18]. Equation (3) is the Ornstein–Uhlenbeck process [33] with the solution

(6) $$f\left(t\right)=f\left(0\right)e^{-\gamma t}+\int_{0}^{t}dt_{1}e^{-\gamma (t-t_{1})}\xi \left(t_{1}\right).$$

For f(0)=0, the correlation time of f(t) is approximately 1/γ, as follows:

(7) $$\begin{array}{ll} \langle f\left(t\right)f\left(t^{\prime }\right)\rangle & =\int_{0}^{t}dt_{1}\int_{0}^{t^{\prime }}dt_{2}e^{-\gamma (t-t_{1})}e^{-\gamma (t^{\prime }-t_{2})}\langle \xi \left(t_{1}\right)\xi \left(t_{2}\right)\rangle \\ & =\frac{D}{\gamma }\left[e^{-\gamma (t^{\prime }-t)}-e^{-\gamma (t+t^{\prime })}\right]\approx \frac{D}{\gamma }e^{-\gamma |t^{\prime }-t|}, \end{array}$$

where we assumed $t^{\prime }>t$ and used Equation (5). Thus, *x* in Equation (3) is driven by the Gaussian noise with the correlation time $\gamma^{-1}$. While the set of Equations (3) and (4) give a PDF in two dimensions (x,f), it is useful to obtain an approximate PDF in the *x* dimension only. To this end, we combine Equations (3) and (4) to obtain the equation for *x* as

(8) $$\partial_{tt}x+\left(\gamma +\partial_{x}g\right)\partial_{t}x=-\gamma g+\xi ,$$

and consider the overdamped limit where $\partial_{tt}x$ is negligible compared with the damping term. This is the so-called unified-colored noise approximation [34], and turns Equation (8) into

(9) $$\left(\gamma +\partial_{x}g\right)\partial_{t}x≃-\gamma g+\xi .$$

We observe that for sufficiently small γ, to O(γ) Equation (9) is, again, an Ornstein–Uhlenbeck process [33] for Q=g+γx:

(10) $$\partial_{t}Q=-\gamma Q+\gamma^{2}x+\xi \approx -\gamma^{}Q+\xi .$$

Thus, the mean value of $\langle Q\left(t\right)\rangle =Q_{0}e^{-\gamma t}$, where $Q_{0}=\langle Q\left(t=0\right)\rangle$, decays exponentially in time while the variance, $\langle {\left(Q-\langle Q\rangle \right)}^{2}\rangle =\frac{1}{2\beta }$, evolves according to

(11) $$\frac{1}{2\beta }=\frac{e^{-2\gamma t}}{2\beta_{0}}+\frac{D(1-e^{-2\gamma t})}{\gamma },$$

where β and $\beta_{0}=\beta \left(t=0\right)$ are the inverse temperatures of p(Q,t) and its initial value, respectively. Therefore, the time-dependent PDF of *Q* is a Gaussian process and is given by

(12) $$p\left(Q,t\right)=\sqrt{\frac{\beta }{\pi }}e^{-\beta {\left(Q-\langle Q\rangle \right)}^{2}},$$

where β is the inverse temperature that satisfies Equation (11).

Since E in Equation (1) and L in Equation (2) are invariant under the change of variables, the Gaussian PDF of *Q* in Equation (12) provides us with a convenient way of calculating them by utilising the property of the Gaussian PDF. Specifically, for the Gaussian PDF of *Q*, E is given by

(13) $$E=\frac{{\left(\partial_{t}\beta \right)}^{2}}{2\beta^{2}}+2\beta {\left(\partial_{t}\langle Q\rangle \right)}^{2},$$

where the first and second terms on the right-hand side are due to the temporal changes in the width and peak position of the PDF. For sufficiently small *D* (large β) and/or large 〈Q〉, E in Equation (13) is dominated by the second term. Furthermore, with a small *D*, Equation (11) becomes $2\beta ∼2\beta_{0}e^{2\gamma t}$. Thus, by substituting $2\beta ∼2\beta_{0}e^{2\gamma t}$, $\partial_{t}\langle Q\rangle =-\gamma Q_{0}e^{-\gamma t}$ into Equation (13), we obtain

(14) $$E∼2\gamma^{2}\beta_{0}Q_{0}^{2},$$

where $Q_{0}=\left(a+\gamma \right)x_{0}+bx_{0}^{3}$, and $x_{0}=\langle x\left(t=0\right)\rangle$ is the mean position of *x* at t=0. To relate Equation (14) to what is observed in the PDF of *x*, we need to find the initial inverse temperature, $\beta_{0}^{x}=1/2\langle {\left(x\left(0\right)-x_{0}\right)}^{2}\rangle$, for p(x,t=0) that corresponds to $\beta_{0}=1/2\langle {\left(Q\left(0\right)-Q_{0}\right)}^{2}\rangle$ (which is the inverse temperature of the PDF of *Q* at t=0). To this end, we use $Q-\langle Q\rangle =\left(a+\gamma \right)x+bx^{3}-\langle \left(a+\gamma \right)x+bx^{3}\rangle ∼\left(x-\langle x\rangle \right)\left(a+\gamma +3b{\langle x\rangle }^{2}\right)$ to leading order for |〈x〉|≫|x−〈x〉| and obtain

(15) $$\langle {\left(Q-\langle Q\rangle \right)}^{2}\rangle ∼\langle {\left(x-\langle x\rangle \right)}^{2}\rangle \left(a+\gamma +3b{\langle x\rangle }^{2}\right){}^{2}.$$

For $x_{0}\gg \gamma ,a$, Equation (15) evaluated at t=0 gives us

(16) $$\beta_{0}∼\frac{\beta_{0}^{x}}{9b^{2}x_{0}^{4}}.$$

Equations (14) and (16) give us

(17) $$E∼\frac{2\beta_{0}^{x}\gamma^{2}x_{0}^{2}}{9b^{2}},\;\;\;L\left(t\right)∼\sqrt{\frac{2\beta_{0}^{x}}{9b^{2}}}\;\gamma x_{0}t.$$

Thus, L(t) increases linearly with time with a slope that is proportional to γ and $x_{0}$ (for small time, small *D*, small γ, and large $x_{0}$). The numerical simulations in Section 4 examine this behaviour in more detail.

Then, by using the conservation of the probability, the time-dependent PDF of *x* is obtained as

(18) $$p\left(x,t\right)=\left|\frac{dQ}{dx}\right|p\left(Q,t\right)=\sqrt{\frac{\beta }{\pi }}\left|\partial_{x}g+\gamma \right|\;\exp\left(-\beta {\left(Q-\langle Q\rangle \right)}^{2}\right).$$

It is interesting to note that p(x,t→∞) in Equation (18) can be either unimodal or bimodal depending on the values of the parameters. This is discussed in detail in Section 3.

Having gained some insight into the leading order behaviour of p(x,t) for small γ, we investigate a more general case of Equation (9). To this end, it is convenient to recast Equation (9) as

(19) $$\partial_{t}x=-\frac{\gamma g}{G}+\frac{1}{G}\xi ,$$

where $G=\partial_{x}g+\gamma$. The corresponding Fokker–Planck equation for p(x,t) is

(20) $$\frac{\partial }{\partial t}p\left(x,t\right)=\frac{\partial }{\partial x}\left[\frac{\gamma g}{G}p\left(x,t\right)\right]+D\frac{\partial }{\partial x}\left[\frac{1}{G}\frac{\partial }{\partial x}\left(\frac{1}{G}p\left(x,t\right)\right)\right].$$

In Equation (20), we used the Stratonovich calculus [33,35,36,37], which recovers the limit of a short correlated forcing from the finite correlated forcing [37]. Although a time-dependent solution to Equation (20) is not easily obtained analytically, a stationary solution can be found and is discussed in detail in Section 3.

## 3. Stationary PDFs

In order to undertake a systematic numerical study in Section 4, we here provide a detailed discussion of stationary PDFs for different parameter values, and determine the parameter space for unimodal versus bimodal PDFs. A stationary PDF found from Equation (18) is

(21) $$p\left(x\right)\propto |G\left(x\right)|\;\exp\left(-\frac{\gamma }{D}\int^{x}g\left(x_{1}\right)G\left(x_{1}\right)dx_{1}\right)=\left|\partial_{x}g+\gamma \right|\;\exp\left(-\frac{\gamma }{2D}\left[g{\left(x\right)}^{2}+2\gamma \int^{x}g\left(x_{1}\right)dx_{1}\right]\right).$$

To O(γ), Equation (21) reproduces Equation (18). To determine the location of the local maxima and minima of p(x) in Equation (21), we calculate

(22) $$\partial_{x}p\left(x\right)=0\;\Rightarrow \;-\frac{\gamma }{D}{\left(\partial_{x}g+\gamma \right)}^{2}g+\partial_{xx}g=0.$$

For $g=ax+bx^{3}$, Equation (22) can be rewritten as

(23) $$x\left[-\frac{\gamma }{D}{\left(a+\gamma +3bx^{2}\right)}^{2}\left(a+bx^{2}\right)+6b\right]=0.$$

Equation (23) gives the solution x=0 and x≠0, indicating the possibility of the bimodal PDF. We then find the non-zero solution by solving

(24) $$-\frac{\gamma }{D}{\left(a+\gamma +3bx^{2}\right)}^{2}\left(a+bx^{2}\right)+6b=0.$$

To this end, it is convenient to make the following three successive changes in variables:

(25) $$\left\{\begin{array}{c} X=a+bx^{2},\\ \alpha ={\left(\Omega +3X\right)}^{2}X, \end{array}\right.\to \;\left\{\begin{array}{c} Y=1+\frac{3X}{\Omega },\\ \frac{3\alpha }{\Omega^{3}}=Y^{2}\left(Y-1\right), \end{array}\right.\to \;\left\{\begin{array}{c} Z=1/Y,\\ Z^{3}+\delta Z-\delta =0, \end{array}\right.$$

with Ω, α, δ defined as

(26) $$\Omega =\gamma -2a,\;\alpha =\frac{6Db}{\gamma },\;\delta =\frac{\gamma {\left(\gamma -2a\right)}^{3}}{18Db}.$$

In order to solve the equation for *Z* in Equation (25), we use the Cardano formula and find the following three roots:

(27) $$Z=\left\{\begin{array}{c} \sqrt[{3}]{\frac{\delta }{2}}\left(S+T\right),\\ \sqrt[{3}]{\frac{\delta }{2}}\left(jS+j^{2}T\right),\\ \sqrt[{3}]{\frac{\delta }{2}}\left(j^{2}S+jT\right). \end{array}\right.$$

Here, $j=-\frac{1}{2}+i\sqrt{\frac{3}{2}}$ and

(28) $$S=\sqrt[{3}]{1+\sqrt{1+\frac{4\delta }{27}}},\;T=\sqrt[{3}]{1-\sqrt{1+\frac{4\delta }{27}}}.$$

Equation (27) gives the non-zero solutions of Equation (24):

(29) $$x_{*}^{2}=\sqrt[{3}]{\frac{4D}{3\gamma b^{2}}}\Psi -\frac{\gamma +a}{3b},$$

where

(30) $$\frac{1}{\Psi }=\left\{\begin{array}{c} S+T,\\ jS+j^{2}T,\\ j^{2}S+jT. \end{array}\right.$$

To find real solutions, we check the discriminant (Δ) of the last equation of Equation (25),

(31) $$\Delta =-27{\left(-\delta \right)}^{2}-4{\left(\delta \right)}^{3}=-4\delta^{2}\left[\frac{27}{4}+\delta \right],$$

as the sign of Δ determines the number of the real root as follows:

- If Δ<0, then one root is real, and two are complex conjugates,
- If Δ=0, then all roots are real, and at least two are equal,
- If Δ>0, then all roots are real and unequal.

From a detailed analysis of different cases provided in Appendix A, we conclude that the existence of a bimodal PDF requires Δ≤0 in Equation (31), and that the peak position of a bimodal PDF is given by

(32) $$x_{*}=\pm \sqrt{\sqrt[{3}]{\frac{4D}{3\gamma b^{2}}}\frac{1}{S+T}-\frac{\gamma +a}{3b}},$$

where

$$\delta =\frac{\gamma {\left(\gamma -2a\right)}^{3}}{18Db},\;S=\sqrt[{3}]{1+\sqrt{1+\frac{4\delta }{27}}},\;T=\sqrt[{3}]{1-\sqrt{1+\frac{4\delta }{27}}}.$$

Finally, a convenient method of identifying parameter values for unimodal versus bimodal PDFs is to check the sign of $\partial_{xx}p\left(x\right)$ at x=0:

(33) $$\partial_{xx}p{|}_{x=0}=\left[6b-\frac{\gamma }{D}{\left(a+\gamma \right)}^{2}a\right].$$

Since a unimodal PDF takes a local maximum at x=0 when $\partial_{xx}p<0$ and a local minimum at x=0 when $\partial_{xx}p>0$, we can see from Equation (33) that a unimodal PDF with $\partial_{xx}p\left(x=0\right)<0$ is more likely for larger γ and smaller *D*. Alternatively, a finite correlation time of *f* (small γ) and a large diffusion (*D*) facilitate the formation of a bimodal PDF.

**Figure 1:**
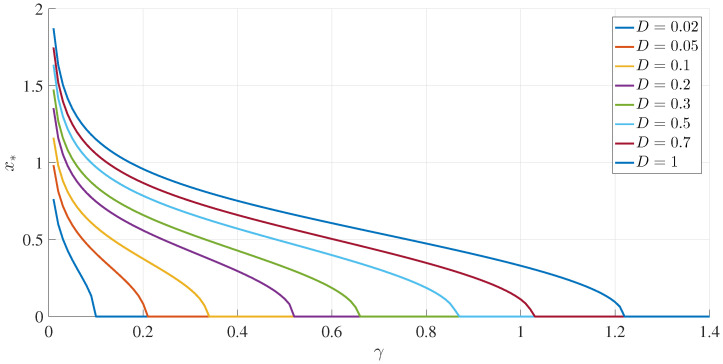
The peak positions ($x_{*}$) as functions of γ, for different values of *D*, as indicated, and a=b=1.

**Figure 2:**
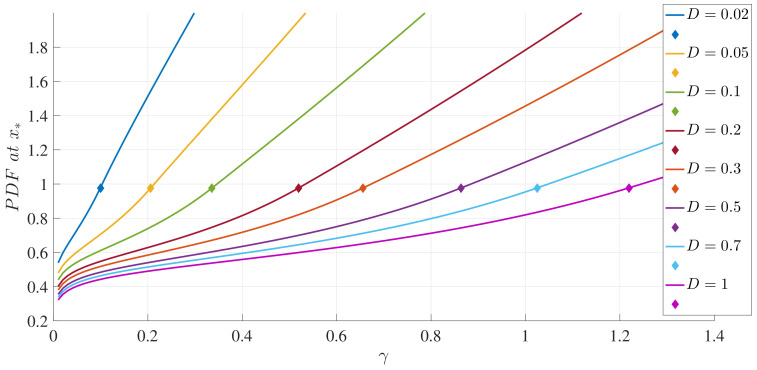
The peak amplitudes ($p(x_{*})$) as functions of γ, for different values of *D*, as indicated, and a=b=1. The small diamonds indicate the transition points between unimodal and bimodal Probability Density Functions (PDFs).

**Figure 3:**
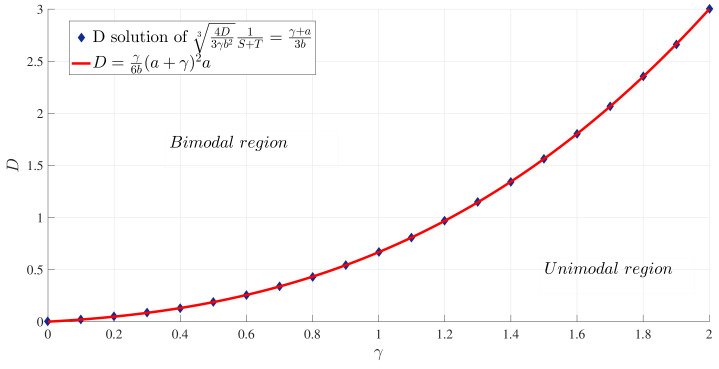
The boundary between unimodal and bimodal PDFs in the parameter space {γ,D}, for a=b=1. The red curve is the solution of $\partial_{xx}p\left(x=0\right)=0$, whereas the blue diamonds are the result of setting R=0.

**Figure 4:**
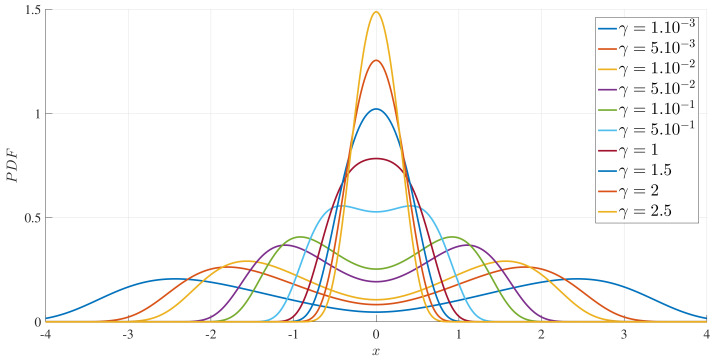
The stationary PDFs for D=0.7, a=b=1, and γ, as indicated. Note the transition between unimodal PDFs for large γ and bimodal PDFs for small γ, in agreement with the boundary shown in Figure 3.

To illustrate these results, Figure 1 and Figure 2 show how the peak position $x_{*}$ and peak amplitude $p(x_{*})$, respectively, vary with γ for a range of *D* values. Figure 3 shows the boundary between the unimodal and bimodal PDFs in the {γ,D} parameter space. These results are for a=b=1, but other values yield the same general boundary shapes, and in particular, the same agreement occurs between the two different evaluation methods, R=0 and (33). The condition $\partial_{xx}p\left(x=0\right)>0$ is therefore a necessary and sufficient condition to have a bimodal PDF. Figure 4 shows what the PDFs look like and how the transition between unimodal and bimodal PDFs comes about.

## 4. Numerical Results

We provided analytical solutions for a time-dependent PDF in certain limiting cases, such as small γ (e.g., Equation (12)), large $x_{0}$ and small time (e.g., Equation (17)) in Section 2, and in the limit of large time, where the PDF settles into a stationary solution, in Section 3. To obtain exact time-dependent solutions to the Fokker–Planck equation (22) for any parameter values, we now use numerical methods in this section and utilise results from Section 3 to perform our numerical simulation systematically. As shown in Appendix B, we can set a=b=1 without any real loss of generality by rescaling the other quantities appropriately. The effective parameter space is therefore reduced to {γ,D}, together with whatever parameters define the initial condition, which we take to be $p\left(x,t=0\right)\propto \exp[-{\left(x-x_{0}\right)}^{2}/10^{-3}]$. That is, $\beta_{0}^{x}=10^{3}$ remains fixed, corresponding to a relatively narrow PDF, and the initial peak position ($x_{0}$) is the one additional parameter. The initial condition (p(x,t=0)) represents the PDF for an initial shear gradient. When the final stationary PDF is unimodal, the mean shear will decrease to zero in the long time limit; when the stationary PDF is bimodal with a peak of $\pm x_{*}$, $x_{0}>x_{*}$ models the relaxation of an initial super-critical gradient ($x_{0}$) to the critical value ($x_{*}$) while $x_{0}<x_{*}$ models the build-up of the gradient from an initial subcritical value to the critical value ($x_{*}$). We are interested in the information change in this relaxation problem and in identifying the difference between the relaxation and build-up of the shear gradient in view of the information change. The numerical implementation of Equation (22) is based on second-order accurate finite-differencing in both *x* and *t*, with up to $10^{4}$ grid points in *x*, and timesteps as small as $10^{-4}$. The domain in *x* is truncated to the interval [−10,10] rather than the original unbounded interval for which the analytic theory applies. As seen in Figure 4, for example, for the parameter values of interest here, the PDFs are well-confined to the interval |x|≤10, making a numerical solution of (22) with boundary conditions of p=0 at x=±10 an excellent equivalent to an infinite interval.

### 4.1. Time Evolution of PDFs

**Figure 5:**
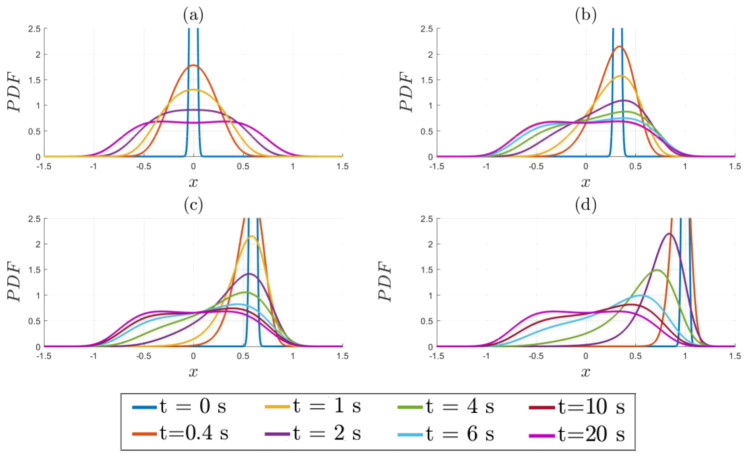
Time evolution of the PDFs for the following initial conditions: (**a**) $x_{0}=0$; (**b**) $x_{0}=0.32$; (**c**) $x_{0}=0.6$; (**d**) $x_{0}=1$. γ=D=1 for all four.

**Figure 6:**
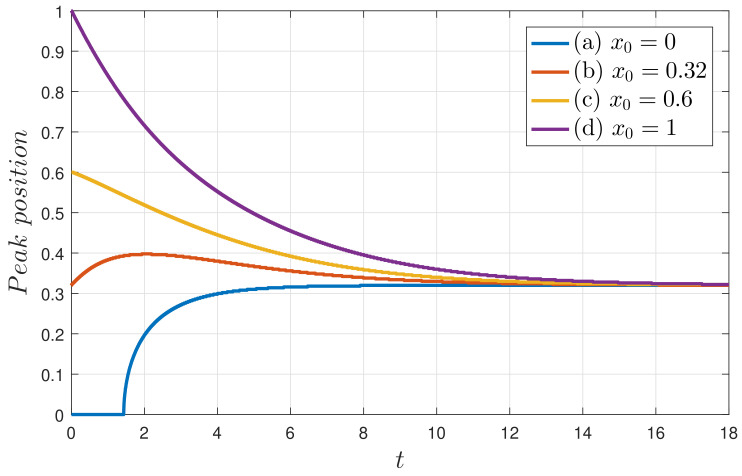
The peak positions of the solutions in Figure 5 as functions of time.

Figure 5 shows examples of how different values of $x_{0}$ ultimately all relax to the same final PDF. Panels (a–d) correspond to $x_{0}=0,\;0.32,\;0.6,\;1$, respectively. γ=D=1, according to Figure 3, is slightly in the bimodal regime, consistent with the final PDF seen here. Figure 6 focuses specifically on how the positions of the peaks evolve in time. Important observations that we can make from Figure 5 and Figure 6 are as follows:

(a) An initial PDF with a peak at $x_{0}=0$ remains unimodal before becoming a bimodal PDF;
(b) An initial PDF with a peak at $x_{0}=x_{*}$ (0.32 for this case) does not maintain the same peak position at $x_{*}$, but moves outward first to $x>x_{*}$ and then inwards to $x_{*}$. This initial outward movement explains why the minimum $L_{\infty }=L\left(t\to \infty \right)$ does not occur for $x_{0}=x_{*}$ in Section 4.2;
(c) An initial PDF with a peak at $x=x_{L}$ (where $x_{L}$ is the $x_{0}$ value which minimises $L_{\infty }$, as defined in Section 4.2) constitutes the border line between different PDF evolutions (an initial PDF with a peak at $x_{0}<x_{L}$ goes outwards and then inwards, while an initial PDF with a peak at $x_{0}>x_{L}$ monotonically moves inwards to $x_{*}$);
(d) An initial PDF with a peak at $x_{0}=1>x_{L}$ monotonically moves inwards.

### 4.2. Information Length: Attractor Structure

**Figure 7:**
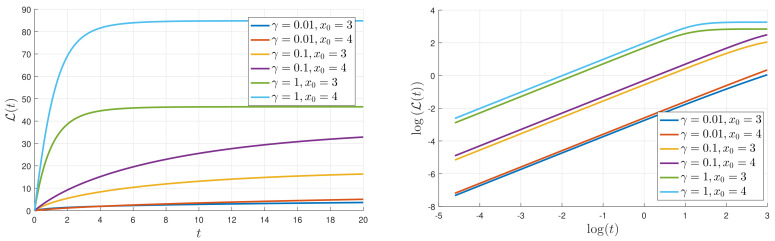
L(t) as a function of *t*, with a linear scale on the left and a logarithmic scale on the right.

**Figure 8:**
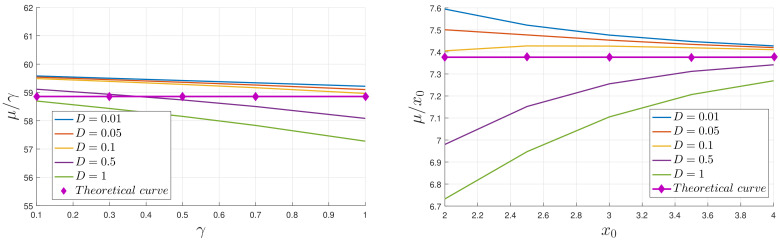
Letting μ denote the numerically computed slope L(t)/t (for small *t*), the left panel shows μ/γ as a function of γ, for $x_{0}=4$, and the right panel shows $\mu /x_{0}$ as a function of $x_{0}$ for γ=0.5. The agreement with the expectations from Equation (17) is seen to be reasonably good.

Since L(t) represents the cumulative change in information, it is zero at t=0 and increases with time. As a PDF settles into a stationary PDF in the limits of a large time, the temporal change in PDFs becomes smaller and then becomes zero, L(t) settling to a constant value of $L_{\infty }\left(x_{0},D,\gamma \right)$. A typical evolution of L(t) is shown in Figure 7 for D=0.5, $x_{0}=3$, and 4, and a range of γ values. The logarithmic scale on the right makes it especially clear that for small times, L grows linearly in time, before eventually equilibrating to its final value, $L_{\infty }$. In order to make more precise comparisons with the analytic prediction (17), Figure 8 shows the results of extracting a numerically computed slope, call it $\mu =\frac{d}{dt}L\left(t\right)$, and compares with the analytic expectation $\sqrt{2\beta_{0}^{x}/9b^{2}}\;\gamma x_{0}$ in Equation (17). That μ is expected to scale linearly with γ and $x_{0}$ and be independent of *D*, is reasonably well reproduced by the numerical data with less than a 10% difference between the theoretical prediction and simulation results (note the small range of the *y*-axis).

**Figure 9:**
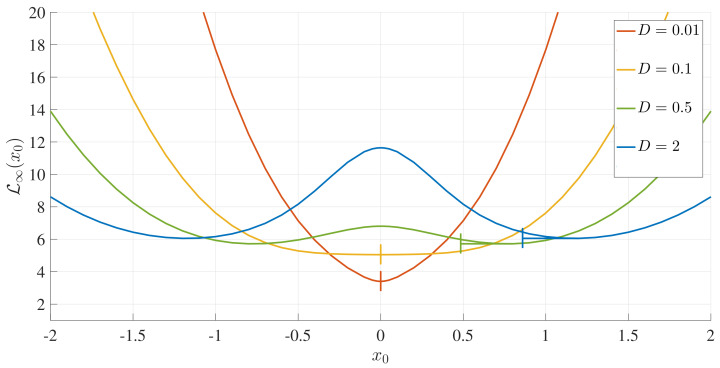
$L_{\infty }$ as a function of $x_{0}$ for *D*, as indicated, and γ=0.5.

$L_{\infty }\left(x_{0},D,\gamma \right)$ is a unique representation of the total number of statistically different states that a PDF evolves through to reach a final unimodal or bimodal PDF. The smaller $L_{\infty }$ is, the smaller the number of states that the initial PDF passes through to reach the final equilibrium. Therefore, $L_{\infty }$ provides us with a path-dependent Lagrangian measure of the distance between a given initial and final PDF. Thus, by choosing a narrow initial PDF at different peak positions ($x_{0}$), we can map out the attractor structure (the proximity of $x_{0}$ to an equilibrium) by measuring $L_{\infty }$ as a function of $x_{0}$. We were particularly interested in how differently $L_{\infty }$ would behave for the final unimodal and bimodal PDFs, which have different stable equilibrium points: x=0 and $x=x_{*}\neq 0$, respectively. To this end, Figure 9 shows $L_{\infty }$ as a function ($x_{0}$) for a range of *D* values. For final bimodal PDFs, the location of the final peak position ($x_{*}$) is shown by a little vertical line.

We note first in Figure 9 that the overall shapes of the curves are drastically different depending on whether the final PDF is unimodal or bimodal. For a unimodal final PDF, the minimum value of $L_{\infty }$ occurs for $x_{0}=0$. This is because $x_{0}=0$ is a stable equilibrium for a unimodal PDF and thus, an initial PDF with the peak ($x_{0}$) closer to x=0 undergoes less change during the evolution of time and is more similar to the final PDF. Therefore, the absolute minimum of $L_{\infty }$ occurs at $x_{L}={\mathrm{argmin}}_{x_{0}}L_{\infty }\left(x_{0}\right)=0$, as can be seen in the orange and yellow curves in Figure 9.

In comparison, x=0 is an unstable equilibrium point for a final bimodal PDF, while $x_{*}\neq 0$, given by Equation (32), is a stable equilibrium point. Therefore, $L_{\infty }$ has a local maximum around $x_{0}=0$ (unstable point). Naively, the minimum value of $L_{\infty }$ would be expected to occur for an initial PDF with $x_{0}=x_{*}$, that is, when the peak position of an initial PDF ($x_{0}$) coincides with that of the final PDF ($x_{*}$). However, the blue and green curves in Figure 9 reveal the very interesting fact that $L_{\infty }$ is actually minimised for $x_{0}=x_{L}>x_{*}$. As noted from Figure 5 and Figure 6, this is because the initial peaks that are sufficiently far away move inwards monotonically, but the initial peaks near $x_{*}$ actually have a more complicated evolution (moving outwards and then inwards).

**Figure 10:**
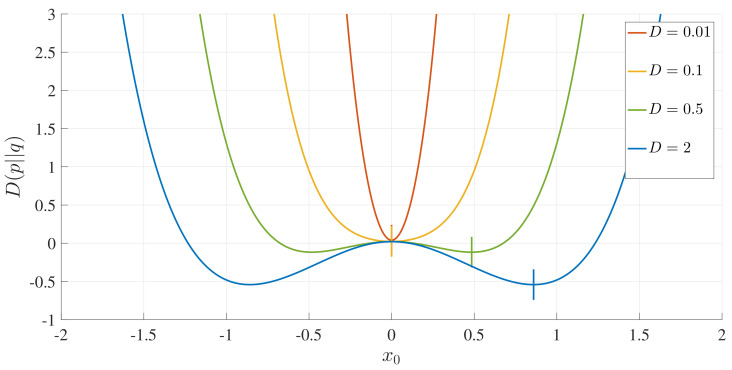
Relative entropy (D(p||q)) as a function of $x_{0}$ for *D*, as indicated, and γ=0.5.

These observations confirm that $L_{\infty }$ is a good Lagrangian measure that captures the attractor structure and dynamics. It is, thus, of particular interest to compare $L_{\infty }$ with the Kullback–Leibler divergence [19] (that is commonly used in comparing PDFs), defined as

(34) $$D\left(p||q\right)=\int p\left(x\right)\ln\left[\frac{p(x)}{q(x)}\right]dx,$$

where p(x) is the initial PDF and q(x) is the final one. Obviously, unlike $L_{\infty }$, D(p||q) depends only on the initial and final PDFs, and thus, does not provide any information on dynamics (e.g., what different states an initial PDF passes through in the time evolution, or how the locations and the shapes of the PDFs evolve in time between initial and final PDFs). Since we have an analytic expression for the stationary PDFs, we computed D(p||q) by numerical integration with the initial PDF used above. Figure 10 shows these results, where the little vertical lines represent the positions of $x_{*}$.

We can see that the absolute minimum relative entropy always occurs when $x_{0}=0$ or $x_{*}$ for unimodal and bimodal PDFs, respectively, unlike $L_{\infty }$. In retrospect, this is not particularly surprising, since the relative entropy only measures the difference between the two PDFs, and an initial PDF located at the final peak position is most similar to the final PDF. Specifically, for a bimodal PDF, the initial PDF at the peak position of the final PDF has the strongest resemblance to the final PDF, with the minimum D(p||q) occurring for $x_{0}=x_{*}$.

**Figure 11:**
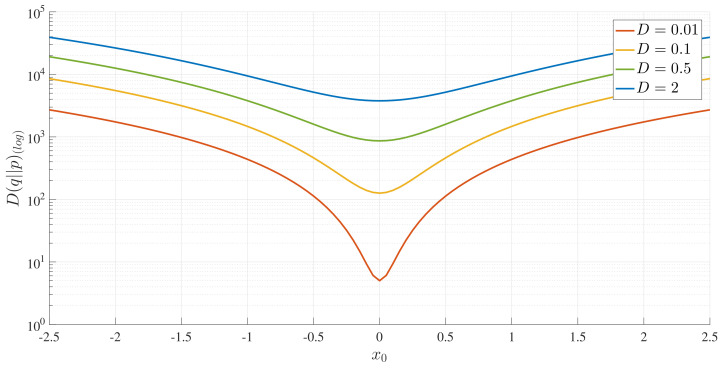
D(q||p) as a function of $x_{0}$ for *D*, as indicated, and γ=0.5.

For completeness, we also show D(q||p) in Figure 11. Unlike Figure 10, the absolute minimum value occurs at $x_{0}=0$, even when the final PDF is bimodal, failing to capture the attractor structure associated with a bimodal PDF. Furthermore, the values of D(q||p) are much larger than those of D(p||q), and thus, a symmetric version ([D(p||q)+D(q||p)]/2) would be dominated by D(q||p). This drastic difference between D(p||q) and D(q||p) calls for care in using symmetric versions.

## 5. Discussion and Conclusions

We investigated the time evolution of PDFs in a toy model of self-organised shear flows using a unified coloured approximation, and utilised the information length to understand information changes and attractor structures. In our model, the formation of shear flows was induced by a finite memory time of a stochastic forcing and was manifested by the emergence of a bimodal PDF, with the two peaks representing non-zero mean values of a shear flow (gradient). We presented a thorough study of PDFs for different correlation time and amplitude values for the stochastic forcing. By solving the Fokker–Planck equation numerically, we investigated the time evolution of PDFs starting with a narrow PDF at different peak positions ($x_{0}$) at time t=0. The cumulative change in information ($L_{\infty }$) beautifully maps out the underlying attractor structures. Specifically, for a unimodal PDF, the minimum value of $L_{\infty }$ occurs for $x_{0}=0$, since $x_{0}=0$ is a stable equilibrium for a unimodal PDF and thus, an initial PDF with a peak ($x_{0}$) closer to x=0 undergoes less change during the time evolution and is more similar to the final PDF; for a bimodal PDF, L is minimised for $x_{0}=x_{L}>x_{*}$, where $x_{*}$ is the peak position of a bimodal PDF. Recalling that $x_{0}$ represents the mean shear gradient at t=0 while $x_{*}$ is a critical shear gradient, $x_{0}=x_{L}>x_{*}$ implies that an initial narrow PDF with a super-critical shear gradient is, in fact, more similar to a final stationary state, while an initial narrow PDF with a mean critical shear gradient undergoes a complicated evolution through the interaction with fluctuations. This is likely to be due to the rapid relaxation of instability at the super-critical state, similar to what was observed in the forward process in the phase transition in [27] (e.g., compare Figure 6b and Figure 7b). That is, a process triggered by instability involves a smaller change in information and thus, a larger change in entropy (as might be expected as a consequence of instability). This reflects a unique property of $L_{\infty }$ which depends on a trajectory/history of a PDF evolution. In comparison, the relative entropy, which only measures the difference between the initial and final PDFs, does not provide any information on the dynamics between the initial and final times. In summary, we demonstrated the importance of studying the dynamics and the merit of the information length in understanding the dynamics and the evolution of PDFs in a toy model of self-organised shear flow. Further work will include the extension of this work to the analysis of our model without unified colored-noise approximation and to other turbulence models, in particular, to quantify the information change associated with intermittency and self-organisation.

## Appendix A. Derivation of Equation (32)

### Appendix A.1. Case $\delta =-\frac{27}{4}\Leftrightarrow \Delta =0$

According to the definitions of *S* and *T* in Equation (28), S=T=1. So, by using $1+j+j^{2}=0$, we calculated Equation (30):

(A1) $$\frac{1}{\Psi }=\left\{\begin{array}{c} S+T=2,\\ jS+j^{2}T=j+j^{2}=-1,\\ j^{2}S+jT=j^{2}+j=-1. \end{array}\right.$$

Consistent with the statement above, we obtained three real solutions. The last two solutions with the same value of $\frac{1}{\Psi }=-1$ in Equation (A1) make Equation (29):

(A2) $$x_{*}^{2}=-\left[\sqrt[{3}]{\frac{4D}{3\gamma b^{2}}}+\frac{\gamma +a}{3b}\right]<0,$$

which is inconsistent, since $x_{*}$ is a real number. On the other hand, the solution $\frac{1}{\Psi }=S+T=2$ can give a consistent solution if $x_{*}^{2}$ in Equation (29) is positive, that is

(A3) $$R\equiv \sqrt[{3}]{\frac{4D}{3\gamma b^{2}}}\frac{1}{S+T}-\frac{\gamma +a}{3b}>0.$$

If not, the PDF is unimodal.

### Appendix A.2. Case $\delta >-\frac{27}{4}\Leftrightarrow \Delta <0$

Δ<0 gives a unique real solution and two complex solutions which are complex conjugates. It is easy to see that the real solution of our interest corresponds to $\frac{1}{\Psi }=S+T$ because $\sqrt{1+\frac{4\delta }{27}}$ is real. Therefore, as long as Equation (A3) holds, we have a bimodal PDF.

### Appendix A.3. Case $\delta <-\frac{27}{4}\Leftrightarrow \Delta >0$

We can take γ<2a, because if γ>2a, then δ>0 (see the last equation in Equation (26)). We thus take a root ($Z_{*}$) of the last equation of Equation (25) with $\delta <-\frac{27}{4}$.

#### Appendix A.3.1. Subcase $Z_{*}>0$

Obviously, in this case,

(A4) $$Y_{*}=\frac{1}{Z_{*}}>0.$$

We recall that

(A5) $$X_{*}=\frac{\gamma -2a}{3}\left(Y_{*}-1\right).$$

Using γ−2a<0 and Equation (A4), we have

(A6) $$X_{*}=\frac{2a-\gamma }{3}\left(1-Y_{*}\right)<\frac{2a-\gamma }{3}<\frac{2}{3}a,$$

which is in contradiction to $X_{*}=a+bx_{0}^{2}>a$. Thus, there is no consistent non-zero solution in this case.

#### Appendix A.3.2. Subcase $Z_{*}<0$

Because $\delta <-\frac{27}{4}<0$, $\delta Z_{*}>0$. Using the last equation in Equation (25), we then have

(A7) $$Z_{*}^{3}-\delta =-\delta Z_{*}<0,$$

and thus,

(A8) $$Z_{*}^{3}<\delta <\frac{-27}{4}\Rightarrow Z_{*}<-\frac{3}{\sqrt[{3}]{4}}.$$

Using Equation (A7) in Equation (A8) then gives us

(A9) $$Z_{*}^{3}-\delta =\delta \left(-Z_{*}\right)<\frac{27}{4}Z_{*}<-\frac{27}{4}\frac{3}{\sqrt[{3}]{4}}.$$

Therefore,

(A10) $$Z_{*}^{3}<\delta -\frac{27}{4}\frac{3}{\sqrt[{3}]{4}}<-\frac{27}{4}\left(1+\frac{3}{\sqrt[{3}]{4}}\right),$$

and

(A11) $$Y_{*}=\frac{1}{Z_{*}}>-\frac{1}{\sqrt[{3}]{\frac{27}{4}\left(1+\frac{3}{\sqrt[{3}]{4}}\right)}}.$$

So, using Equation (A11) in Equation (A5) gives us

(A12) $$X_{*}=\frac{2a-\gamma }{3}\left(1-Y_{*}\right)<\frac{2a-\gamma }{3}\left[1+\frac{1}{\sqrt[{3}]{\frac{27}{4}\left(1+\frac{3}{\sqrt[{3}]{4}}\right)}}\right]<\frac{2}{3}\left[1+\frac{1}{\sqrt[{3}]{\frac{27}{4}\left(1+\frac{3}{\sqrt[{3}]{4}}\right)}}\right]a≃0.91a.$$

Equation (A12) is in contradiction with $X_{*}=a+bx_{*}^{2}>a$. Therefore, there is no consistent solution in this case. This proves that in the case Δ>0, there is no consistent non-zero solution ($x_{*}$) for a bimodal PDF.

### Appendix A.4. Summary

From the analyses above, we can conclude that the existence of a bimodal PDF requires Δ≤0 and

(A13) $$R\equiv \sqrt[{3}]{\frac{4D}{3\gamma b^{2}}}\frac{1}{S+T}-\frac{\gamma +a}{3b}>0.$$

To make sure that this solution in Equation (29) corresponds to a local maximum, we need to show $\partial_{xx}p\left(x=x_{*}\right)<0$. To this end, we recall that $x_{*}$ satisfies Equation (23)

(A14) $$-\frac{\gamma }{D}{\left(\partial_{x}g\left(x_{*}\right)+\gamma \right)}^{2}\frac{g(x_{*})}{x_{*}}+6b=0.$$

We then find the second derivative of the stationary PDF at $x_{*}$, as follows:

(A15) $$\begin{array}{c} \partial_{xx}p\left(x_{*}\right)\exp\left(\frac{\gamma }{2D}[g{\left(x_{*}\right)}^{2}+2\gamma \int^{x_{*}}g\left(x_{1}\right)dx_{1}]\right)=[-\frac{2\gamma }{D}\left(\partial_{x}g+\gamma \right)g\partial_{xx}g\\ -\frac{\gamma }{D}{\left(\partial_{x}g+\gamma \right)}^{2}\partial_{x}g+6b+\left[-\frac{\gamma }{D}g\partial_{x}g\right]\left[-\frac{\gamma }{D}{\left(\partial_{x}g+\gamma \right)}^{2}g+\partial_{xx}g\right]{]}_{x=x_{*}}. \end{array}$$

By using Equation (23), we see that the last term in Equation (A15) is identically zero. Furthermore, using Equation (A14), we simplify Equation (A15) as

(A16) $$\begin{array}{c} \partial_{xx}p\left(x_{*}\right)\exp\left(\frac{\gamma }{2D}[g{\left(x_{*}\right)}^{2}+2\gamma \int^{x_{*}}g\left(x_{1}\right)dx_{1}]\right)\\ ={\left[\frac{\gamma }{D}\left(\partial_{x}g+\gamma \right)\left[-2g\partial_{xx}g+\left(\partial_{x}g+\gamma \right)\left(\frac{g}{x}-\partial_{x}g\right)\right]\right]}_{|x=x_{*}}\\ =-\frac{\gamma }{D}\left(a+\gamma +3bx_{*}^{2}\right)\left[12b\left(ax_{*}^{2}+bx_{*}^{4}\right)+2x_{*}^{2}\left(a+\gamma +3bx_{*}^{2}\right)\right]. \end{array}$$

Since the exponential is positive, $\partial_{xx}p\left(x_{*}\right)<0$ in Equation (A16), confirming a local maximum of a PDF at $x=x_{*}$.

## Appendix B. Rescaling

We show, in detail, how we rescale Equations (3) and (4) to make a=b=1. We first rescale *x* by using $x=\sqrt{\frac{a}{b}}\tilde{x}$ in Equations (3) and (4) to recast them as

(A17) $$\left\{\begin{array}{c} \partial_{t}\tilde{x}=\sqrt{\frac{b}{a}}\left[-\frac{a\sqrt{a}\tilde{x}}{\sqrt{b}}-\frac{a\sqrt{a}{\tilde{x}}^{3}}{\sqrt{b}}+f\right],\\ \partial_{t}f=-\gamma f+\xi , \end{array}\right.$$

and thus,

(A18) $$\left\{\begin{array}{c} \partial_{t}\tilde{x}=-a\left(\tilde{x}+{\tilde{x}}^{3}\right)+\sqrt{\frac{b}{a}}f,\\ \partial_{t}f=-\gamma f+\xi . \end{array}\right.$$

Next, we rescale *t* by using $t=\frac{\tilde{t}}{a}$

(A19) $$\left\{\begin{array}{c} \partial_{\tilde{t}}\tilde{x}=-\tilde{x}-{\tilde{x}}^{3}+\frac{1}{a}\sqrt{\frac{b}{a}}f,\\ \partial_{\tilde{t}}f=-\frac{\gamma }{a}f+\frac{\xi }{a}. \end{array}\right.$$

We let $\tilde{f}=\frac{1}{a}\sqrt{\frac{b}{a}}f$

(A20) $$\left\{\begin{array}{c} \partial_{\tilde{t}}\tilde{x}=-\tilde{x}-{\tilde{x}}^{3}+\tilde{f},\\ \partial_{\tilde{t}}\tilde{f}=-\frac{1}{a}\gamma \tilde{f}+\frac{1}{a^{2}}\sqrt{\frac{b}{a}}\xi . \end{array}\right.$$

We then let $\tilde{\gamma }=\frac{1}{a}\gamma$ and $\tilde{\xi }=\frac{1}{a^{2}}\sqrt{\frac{b}{a}}\xi$

(A21) $$\left\{\begin{array}{c} \partial_{\tilde{t}}\tilde{x}=-\tilde{x}-{\tilde{x}}^{3}+\tilde{f},\\ \partial_{\tilde{t}}\tilde{f}=-\tilde{\gamma }\tilde{f}+\tilde{\xi }. \end{array}\right.$$

Finally, we rescale $\langle \tilde{\xi }\left(t\right)\tilde{\xi }\left(t^{\prime }\right)\rangle =\frac{b}{a^{5}}\langle \xi \left(t\right)\xi \left(t^{\prime }\right)\rangle =\frac{b}{a^{5}}D\delta \left(t^{\prime }-t\right)=\frac{b}{a^{4}}D\delta \left({\tilde{t}}^{\prime }-\tilde{t}\right)=\tilde{D}\delta \left({\tilde{t}}^{\prime }-\tilde{t}\right)$.
